# Microfluidic investigation of pore-size dependency of barite nucleation

**DOI:** 10.1038/s42004-023-01049-3

**Published:** 2023-11-16

**Authors:** Jenna Poonoosamy, Abdulmonem Obaied, Guido Deissmann, Nikolaos I. Prasianakis, Moritz Kindelmann, Bastian Wollenhaupt, Dirk Bosbach, Enzo Curti

**Affiliations:** 1 Institute of Energy and Climate Research (IEK-6): Nuclear Waste Management, Forschungszentrum Jülich GmbH, 52425 Jülich, Germany; 2 Laboratory for Waste Management, Paul Scherrer Institut, CH-5232 Villigen PSI, Switzerland; 3 Ernst Ruska-Centre for Microscopy and Spectroscopy with Electrons (ER-C 2): Materials Science and Technology, Forschungszentrum Jülich GmbH, 52425 Jülich, Germany; 4 Institute of Bio- and Geosciences (IBG-1): Biotechnology, Forschungszentrum Jülich GmbH, 52425 Jülich, Germany

**Keywords:** Geochemistry, Chemical engineering, Nuclear waste, Chemical safety

## Abstract

The understanding and prediction of mineral precipitation processes in porous media are relevant for various energy-related subsurface applications. While it is well known that thermodynamic effects can inhibit crystallization in pores with sizes <0.1 µm, the retarded observation of mineral precipitation as function of pore size is less explored. Using barite as an example and based on a series of microfluidic experiments with well-defined pore sizes and shapes, we show that retardation of observation of barite crystallite can already start in pores of 1 µm size, with the probability of nucleation scaling with the pore volume. In general, it can be expected that mineralization occurs preferentially in larger pores in rock matrices, but other parameters such as the exchange of the fluids with respect to reaction time, as well as shape, roughness, and surface functional properties of the pores may affect the crystallization process which can reverse this trend.

## Abstract (web summary)

Mineral precipitation in porous media is relevant for various energy-related applications such as oil extraction, geothermal systems, or nuclear waste disposal, but crystallization retardation as a function of pore size and shape is poorly explored. Here, the authors study barite crystallization in micro-confinement, showing that retardation of crystallization can start in pores of less than 1 µm in size, with the probability of nucleation scaling with pore volume.

## Introduction

The first step of the crystallization of a mineral in water-filled pores or on mineral surfaces, i.e., nucleation, strongly affects the further structural evolution of the pore space and consequently the hydraulic pathways in a rock matrix^1–4^. Barite (BaSO_4_) is known to crystallize during oil and gas extraction, reducing the permeability of reservoir rocks and consequently decreasing the extraction efficiency. In deep geological repositories for nuclear waste, barite is an important mineral for the retention of radium and other toxic elements^5–9^. During wastewater treatment from hydraulic fracturing, (Ra,Ba)SO_4_ solid solutions are a major component of TENORM wastes with important environmental consequences^10^. Understanding the nucleation of barite, elucidating the pore size dependency, and quantifying thermodynamic and kinetic parameters are a prerequisite for the fine-tuning of these systems or to provide robust predictions of the subsurface evolution^11–14^.

The most common framework to rationalize nucleation is the classical nucleation theory (CNT), which describes a first-order phase transformation from a metastable phase, whereby the free energy of a nascent nucleus drives nucleation. The change in free energy, $\Delta G$, associated with the formation of a nucleus of radius, *r*, in free water (i.e., homogeneous nucleation, HOM) is given as follows^15^:

1 $$\Delta G({{{{{{\rm{HOM}}}}}}})=\Delta {G}_{{{{{{\rm{bulk}}}}}}}+\Delta {G}_{{{{{{\rm{surface}}}}}}}=-\frac{4}{3}\pi {r}^{3}\Delta g+4\pi {r}^{2}\gamma$$

where $\Delta g$ [J m^−3^] is the change is the free energy change Gibbs energy potential per unit volume associated with the change of a molecule in solution to the bulk solid and $\gamma$ [J m^−2^] is the interfacial free energy between nascent nucleus and aqueous phase. This equation describes the increase in free energy of the nucleus caused by the work necessary to develop its surface area. The increase in free energy reaches a maximum (Δ*G*_c_) when the nucleus reaches a critical size, beyond which further crystal growth is favorable. Nuclei smaller than the critical size are unstable and will dissolve, while nuclei exceeding the critical size via statistic fluctuations will grow spontaneously and start the precipitation process.

Nucleation on a surface, usually at defect sites and other regions where the energetics differ from the bulk matrix, is termed heterogeneous nucleation (HET) and the associated change in Gibbs energy associated for a nucleus on a surface is given as the product of the $\Delta G({{{{{{\rm{HOM}}}}}}})$ and the function of the contact angle $({{\uptheta }})$ between the precipitating mineral and the substrate:

2 $$\Delta G({{{{{\rm{HET}}}}}})=\Delta G({{{{{\rm{HOM}}}}}})\Psi (\uptheta )\;{{{{{\rm{with}}}}}}\;\Psi (\uptheta )=\left(\frac{1}{4}\right)(2+{{{{{\rm{cos}}}}}}\uptheta ){(1-{{{{{\rm{cos}}}}}}\uptheta )}^{2}$$

CNT was originally derived for the formation of nuclei from supersaturated water vapor. It was later extended to the nucleation of crystals from solution with several assumptions, e.g., that the interfacial energy between the nucleus and the solution is the same as that of the bulk mineral, or that the critical nucleus is a spherical entity with a sharp interface, which is true for a vapor–liquid interface but not always the case for solid-fluid interfaces^16^. In the last decades, alternative nucleation pathways have been discussed. In contrast to CNT, after which a given solid nucleates from solution directly with its final lattice structure, non-classical nucleation requires formation of water-rich intermediates, so-called pre-nucleation clusters^17,18^. Pre-nucleation clusters (PNCs) play a role in the nucleation process by engaging in a mechanism involving the separation of liquid phases through the aggregation of monomers. After aggregation, these clusters undergo a process of dehydration and compaction within a secondary liquid phase, ultimately resulting in the formation of hydrated amorphous solid phase^19^ which dissolves in favor of crystalline phases^20^. This description applies, e.g., to the crystallization of gypsum^21^. Although recent advances involving cryo-electron emission tomography or synchrotron-based micro XRD support this hypothesis for a number of geochemical systems of interest^22,23^, particularly calcium carbonates, the non-classical nucleation pathways remain debated, and CNT remains widely used^24^.

Barite crystallization has been extensively studied in classical batch experiments, where the ratio of water to solid is large. However, in compacted rock matrices, crystallization occurs either directly from the aqueous phase in the small water-filled pore volumes (HOM) or on the mineral surface of the pores (HET)^25^. Due to the comparatively low fluid velocities in geosystems (compared to flow rates in laboratory crystallizers), the smaller water volumes, the properties and characteristics of the nearby mineral surfaces, the structure of water on their surface in nano-confinement and the nucleation mechanism, the subsequent crystallization in (tight) rock matrices (porous media) occurs differently than in bulk solution^1,24^. There is evidence, based on various observations, that nucleation is inhibited in small pores, which is explained by two distinct effects that may act in combination: a thermodynamic effect and a kinetic effect^24^.

Thermodynamic hindrance to nucleation arises from the increase in solubility induced by the confining pressure on growing crystals trapped in small pores (crystallization pressure). According to Rijniers et al.^26^, the logarithm of the solubility of a given anhydrous solid increases proportionally to the confining pressure (cf. Eq. S1.3 and Fig. S1 in Supplementary Note 1). This purely thermodynamic effect is well-known under the term “pressure solution”^27^ or “pore size controlled solubility” (PCS)^28^. In isotropic porous media, this mechanism results in preferential mineral precipitation in larger pores, due to the lower pressure experienced by the solid particles (and hence lower solubility) in these pores compared to smaller pores, where crystallization leads to locally high crystallization pressure^28^. Further evidence for such an effect comes also from experiments showing preferential crystallization in macro-pores (100 µm) over nanopores (8–31 nm)^29^ and field observations indicating preferential mineralization in larger pores^30^.

Although nucleation itself is not a kinetic process, solutions can remain supersaturated without the formation of precipitates for some time, delaying the thermodynamic equilibration process, introducing a “kinetic effect” in the system. This “kinetic effect” or retardation in the formation of crystallites ultimately arises from the fact that unlike in free bulk solution, a porous medium divides the fluid into many pores, which act as independent volumes for nucleation^31^. Because the nucleation probability is proportional to the volume of the solution, the nucleation rate is inversely proportional to the volume, and precipitation in small pores is most likely retarded compared to a bulk solution. Counter diffusion experiments leading to crystallization in silica-gel (porous media) have shown retardation of nucleation in smaller pores^31^. However, in these counter-diffusion experiments, there was a lack of control over the individual size and shape of the pores and over the evolving diffusive properties, due to precipitation in the silica-gel, which also had feedback on the precipitation processes. It is also noted that within the same porous medium, pores of very different volume can exist. The preferential flow and mass transport paths typically include the larger pores (lower resistivity), while the smaller pores might be less accessible in terms of mass transport of reactants and products. Contributions of such mechanisms are very difficult to quantify and deconvolute when realistic systems are studied.

Although many studies converge towards inhibition of nucleation in small pores, according to the latest reviews on crystallization in confinement, it is not possible to make this a generality. For example, Hedges and Whitelam^32,33^ conducted a 2D simulation in nano-meter size capillaries using the Ising model and observed that the filling rate of a capillary by a mineral precipitate increases when the width of the capillary was ~1.5 times bigger than the size of a critical nucleus. The authors concluded that the specific size and shape of a pore could lower the free energy barrier for nucleation to occur.

In porous media, where solute transport and crystallization processes are coupled and influence each other, it is difficult to conclude based on post-experimental analyses or natural systems^4^. However, the development of methodologies with in situ characterization provides a promising approach to unravel hydrogeochemically coupled nucleation processes^30,34,35^. In this study, we conducted experiments monitored by time resolved microscopy to investigate barite nucleation in confinement. Our investigations included several steps to study the processes in isolation and allow us to focus on specific mechanisms. In the first step, a bulk experiment combining optical imaging and time-resolved Raman spectroscopy showed that nucleation of barite occurs without any amorphous precursor phase. In a second step, experiments in nano-confined volumes of solution were conducted. The advantage of this approach is that it allows to conduct hundreds of isolated experiments in parallel within the same microfluidic chip, each experiment within one nano-droplet reactor. These microfluidic experiments, in which droplets of supersaturated aqueous solutions ranging between 0.3 nL (diameter ~80 µm) and 15 nL (diameter ~400 µm) in volume were generated in an oil carrier fluid, showed that nucleation scales with the volume of fluid as predicted by CNT. While our statistical analysis showed that barite nucleation will be significantly retarded in pores <1 µm, the theoretical calculations showed that the PCS effect would become effective only in pores of sizes less than 0.1 µm. In a third step, the influence of diffusive transport was investigated by inducing the crystallization of barite in a pore network consisting of large and small micrometer-sized pores with circular cross-section interconnected by fine squared capillaries with micrometric aperture. Although preferential mineralization was generally observed in the larger circular pores, at low supersaturation (SI < 2.2) crystallization was observed only within the fine interconnecting capillaries. This unexpected behavior is possibly a consequence of the high reactive surface area (pitches) per unit solution volume in the capillaries compared to the circular pores, which may increase the density of nucleation sites per unit solution volume in the capillaries. In nano-porous media, the crystallization process is mainly controlled by the thermodynamic pore size effect, while in micro-porous media geometry effects and heterogeneous nucleation kinetics driven by the surface energy of the substrate play a major role. Consequently, it can be expected that mineralization occurs preferentially in larger pores in rock matrices but other parameters such as (i) exchange of the fluids w.r.t. reaction time, and (ii) the shape, roughness, and surface functional properties of the pore substrate should also be considered as they might obscure or reverse this trend.

## Results

### Crystallization pathway of barite

**Fig. 1:**
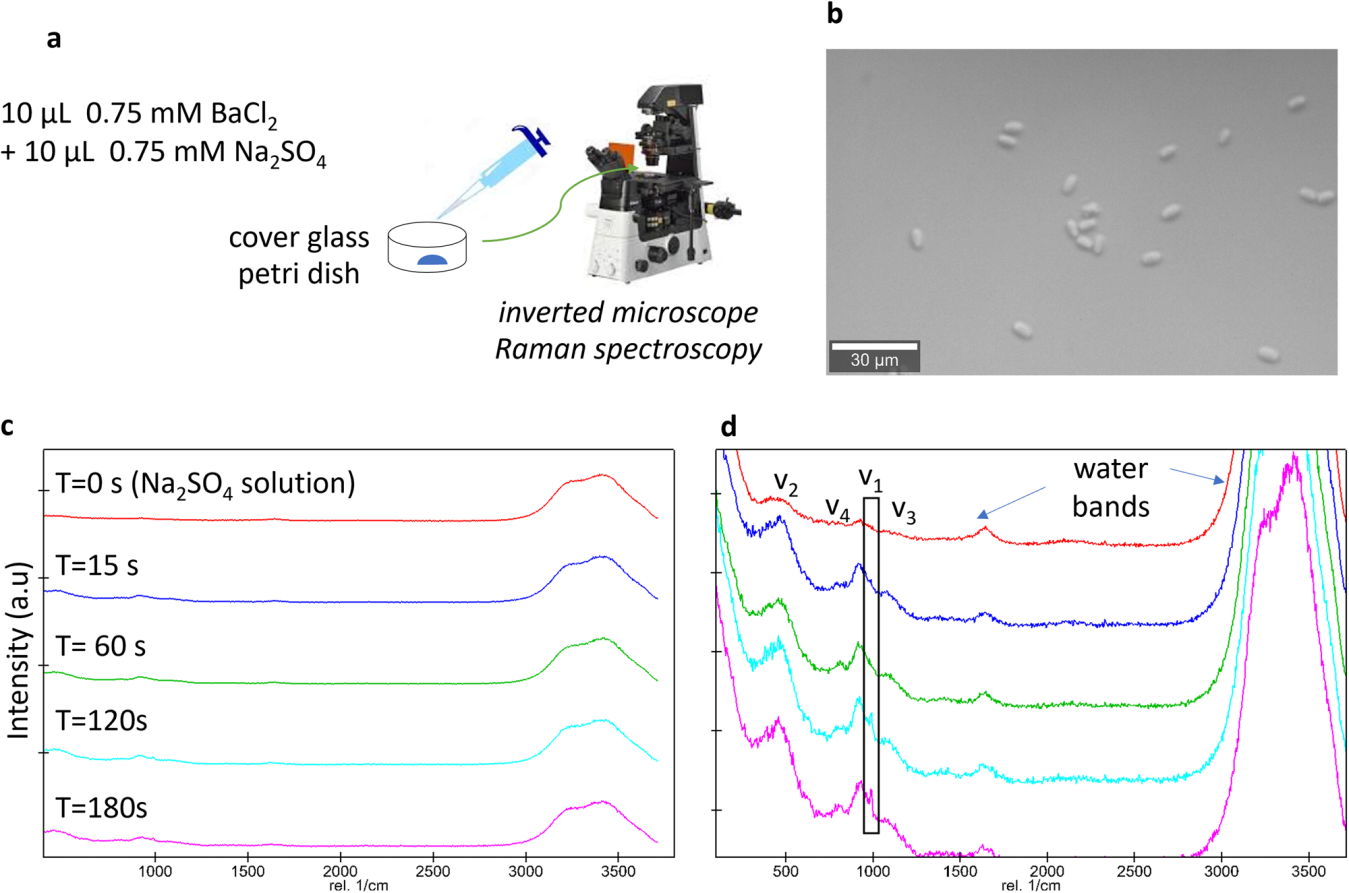
Collection of datasets from the pseudo bulk experiment with SI_(initial)_ = 2.8. **a** Experimental setup; **b** optical micrograph of barite crystals after three minutes; **c** Raman spectra collected during the experiment; **d** zoom on the intensity of the SO_4_^2^^−^ signature bands with *ν*_1_ and *ν*_3_ corresponding to the symmetric and the anti-symmetric stretching mode respectively, and *ν*_2_ and *ν*_4_ corresponding to the bending vibrations^3^. The water bands indicated refer to the H-O-H bending mode at 1648 cm^−1^, and the symmetric and anti-symmetric O-H bands at 3280 and 3410 cm^−1^, respectively.

To investigate its crystallization pathway, barite was allowed to precipitate from an unconfined bulk solution in a cover glass bottom petri dish according to Eq. 3. The initial saturation index (SI, common logarithm (log_10_) of the ion activity product over the solubility product^36^) was 2.8 and the process was monitored by optical microscopy (Fig. 1a).

3 $${{{{{{{\rm{Ba}}}}}}}}_{({{{{{\rm{aq}}}}}})}^{2+}+{{{{{{{\rm{SO}}}}}}}}_{4({{{{{\rm{aq}}}}}})}^{2-}\leftrightarrow {{{{{{{\rm{BaSO}}}}}}}}_{4({{{{{\rm{s}}}}}})}$$

Barite crystallites were observed within 30 seconds and grew with time to form rod-shaped crystals as shown in Fig. 1b. The Raman measurements during the experiments showed the signature of pure barite from an early stage (Fig. 1c) with the prominent *ν*_1_ (990 ± 5 cm^−1^) bands already present 15 s after BaCl_2_ addition, implying that barite nuclei were already present before the first detectable crystals. The intensity of the *ν*_1_ band increased with time. In this bulk experiment, nucleation took place within seconds. No amorphous phases nor shifts and enlargements of Raman bands^20^ as commonly reported for mineral crystallization via multi-step nucleation^37^ were observed here. Additional measurements collected over a longer measuring time (documented in Fig. S2 in Supplementary Note 2) also showed similar results.

Nucleation occurred both on the surface of the cover glass but also in the free solution. The latter cannot be explained by homogeneous nucleation, since this nucleation mechanism would require much longer induction time compared to the observed one. One potential explanation for this observation would be heterogeneous nucleation on impurity particles with sizes less than the filter size, as suggested by previous crystallization studies^24^. For barite, it is unlikely that homogeneous nucleation occurs at an SI of 2.8 given the highly competitive heterogeneous nucleation rate versus the homogeneous rate^25^ (see also calculated nucleation rates in next section).

The current observations regarding the barite nucleation pathway is in line with previous X-ray absorption experiments^38^ and are consistent with direct nucleation of crystalline barite according to a CNT mechanism. However, we cannot exclude the formation of short-lived amorphous precursors, as observed by TEM and FTIR^39^ during barite nucleation. In the further evaluation of our results, we will therefore discuss the capabilities of CNT to describe our experimental observations.

### Barite nucleation in confinement

**Fig. 2:**
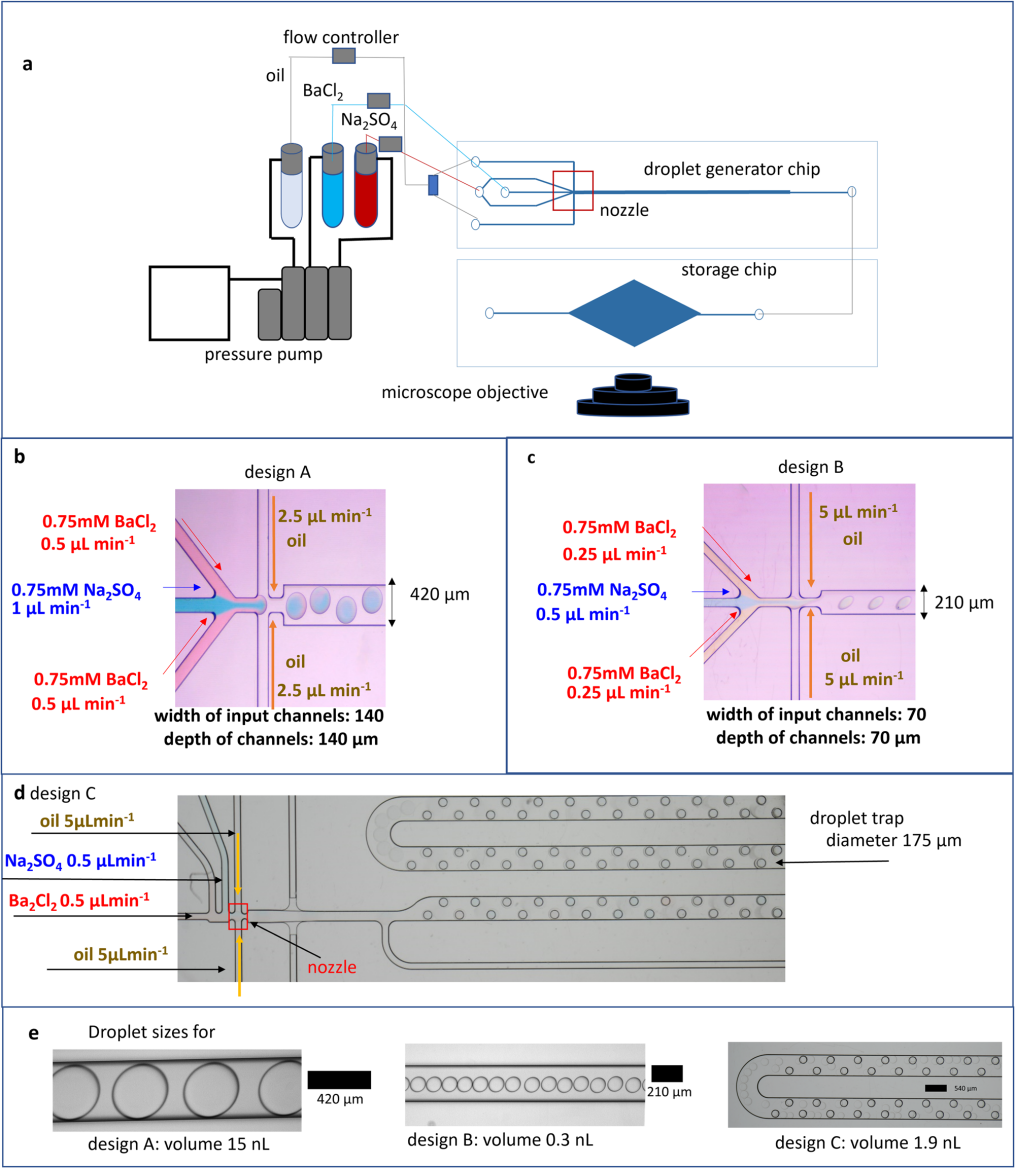
Experimental setup and chip design for droplet generation. **a** Nano volume droplet generation setup with a pump injecting a dispersed phase (reacting aqueous solutions) and a continuous phase (oil phase) into a droplet generator chip connected to a droplet storage chip; **b**, **c** dimensions of the chips with zoom on the nozzle i.e., the mixing region of the carrier and dispersed phase for designs A and B, respectively; **d** chip design C; **e** sizes and volumes of droplets generated by chip designs A–C.

**Fig. 3:**
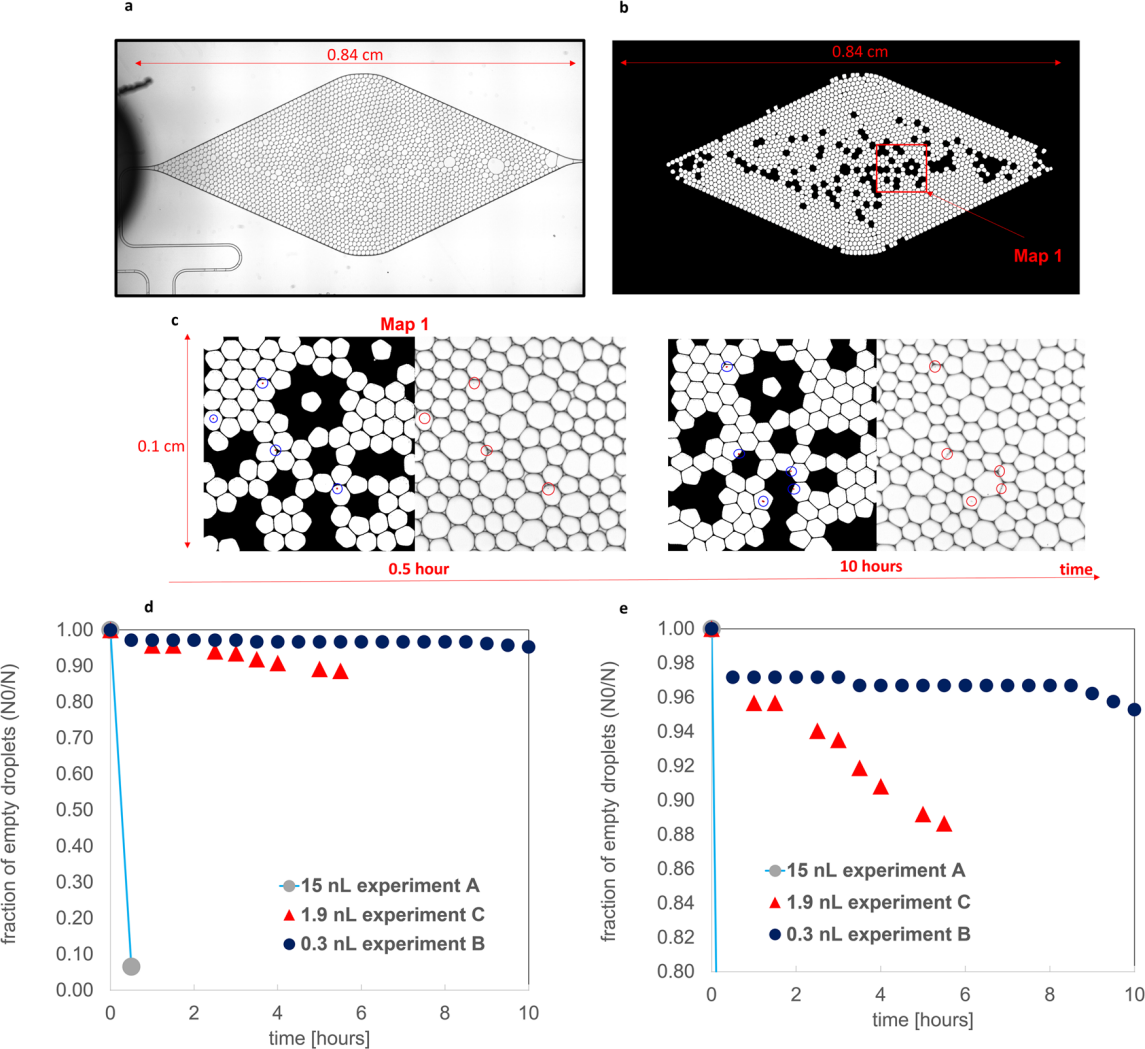
Processing of image datasets from droplet microfluidic experiments to extract the probability of nucleation and the rate. **a** Micrograph of the storage chip of experiment B after 30 min; **b** segmented image with droplets of larger or smaller volumes, resulting from instabilities in the flow, removed; **c** micrographs (right) and corresponding segmented images (left) of a sample location map after 0.5 and 10 h, respectively, with the red circles indicating the presence of crystallites; **d** graph showing the fraction of crystallite-free droplets as a function of reaction time for experiments A–C with **e** showing a change in the range of the *y*-axis to enhance visualization.

Barite nucleation was studied in nL-size droplets that were generated by a microfluidic droplet generator chip and were subsequently stored for monitoring by optical microscopy. At the time of formation of the nanodroplets, the SI was set uniformly to SI = 2.8 with equimolar Ba and SO_4_ concentrations as in the bulk experiment. The frequency of barite nucleation events occurring within the droplets collected in the storage chip (experiments A and B; Fig. 2a–c) or the storage platform (experiment C; Fig. 2d), respectively, was monitored. An example of the micrographs collected is shown in Fig. 3a with a zoom on the number of crystallites that appeared after 30 min and 6 h of reaction time, respectively (Fig. 3b). These images were segmented and processed to extract the fraction of droplets with and without any crystallite during the time evolution of the experiment. In experiment A (15 nL droplet), nucleation was observed in 99.4% of droplets after 30 min, with 2–3 crystallites per droplet. In contrast, only 5% and 15% of the 212 and 182 droplets analyzed in experiments B (0.3 nL droplet) and C (1.9 nL droplet), respectively, contained a single crystallite after 6 h (Fig. 3c). Our results show an unambiguously distinct decrease in the probability of nucleation events in the droplets as their volume decreases.

Assuming that nucleation occurs continuously and independently at a steady nucleation rate, *J* [m^−3 ^s^−1^], the cumulative probability, ${P}_{0}\left(t\right)$, that nucleation has not occurred in a volume, *V*, at time *t* is given by the exponential failure distribution^24,40^.

4 $${P}_{0}\left(t\right)={e}^{-{JVt}}\approx \frac{{N}_{0}}{N}$$

For a given total number of droplets, *N*, the probability ${P}_{0}\left(t\right)$ approximates the fraction of droplets that do not contain crystals, *N*_0_/*N*^41^, where *N*_0_ are the number of droplets without nucleation. Using Eq. 4, a higher nucleation rate for experiment A at 2.2 × 10^6 ^m^−3 ^s^−1^ was calculated (considering the first nucleation event only). The nucleation rates for experiments B and C, where the flow rates of the continuous and dispersed phase were similar, and lower than in experiment A (2.2 × 10^4^ and 1.8 × 10^4 ^m^−3^ s^−1^ respectively, averaging to (2.0 ± 0.2) × 10^4 ^m^−3^ s^−1^). The higher nucleation rate in experiment A can be explained by higher fluid velocities (generated by the higher injection rates of the aqueous fluid in this experiment) in the microfluidic channels (at the nozzle), which enhances mixing of solutes in the droplets and therefore increases the probability of nucleation^24^.

To decipher the nucleation process (homogeneous (HOM) versus heterogeneous (HET)) in the droplet experiments, we calculated the theoretical nucleation rate (Eq. 5) for both mechanisms, which can be derived from the free energy barrier described by CNT (Eqs. 1 and 2).

5 $$J=\Gamma \exp \left(-\frac{\Delta {G}_{c}}{{kT}}\right)$$

where *k* is the Boltzmann constant (1.380649.10^−23 ^J K^−1^), *T* is the absolute temperature (298.15 K), Γ a pre-exponential factor (see Supplementary Note 3 for details) and Δ*G*_c_ is the free energy associated with the formation of a nucleus of critical size.

This introduces a time dependency (rate) in the overall reactive transport process, which is by definition a kinetic aspect. As noted earlier, this kinetic effect is an indirect consequence of the nucleation process driven by pore size dependency^15^. The rates for homogeneous nucleation at a SI of 2.8 (experimental conditions), heterogeneous nucleation on fairly good wetting surface (contact angle 80°), and heterogeneous nucleation on a good wetting surface (contact angle 30°) (see detailed analyses in Fig. S3 and Table S1 in Supplementary Note 3) were evaluated using Eq. 5 at J_HOM = 6.7 × 10^−14^, J_HET80° = 2.8 × 10^0^, and J_HET30° = 6.5 × 10^15 ^m^−3^ s^−1^, respectively. Therefore, the moderately high measured nucleation rate of (2.0 ± 0.2) × 10^4 ^m^−3^ s^−1^ in the droplet experiments apparently excludes homogeneous nucleation in favor of heterogeneous nucleation on less hydrophilic surfaces (contact angle 72.9°), which could be provided by unavoidable fine impurity particles present in the solution, which can act as active centers for nucleation^42^.

### Effect of transport on nucleation processes under micro-confinement

**Fig. 4:**
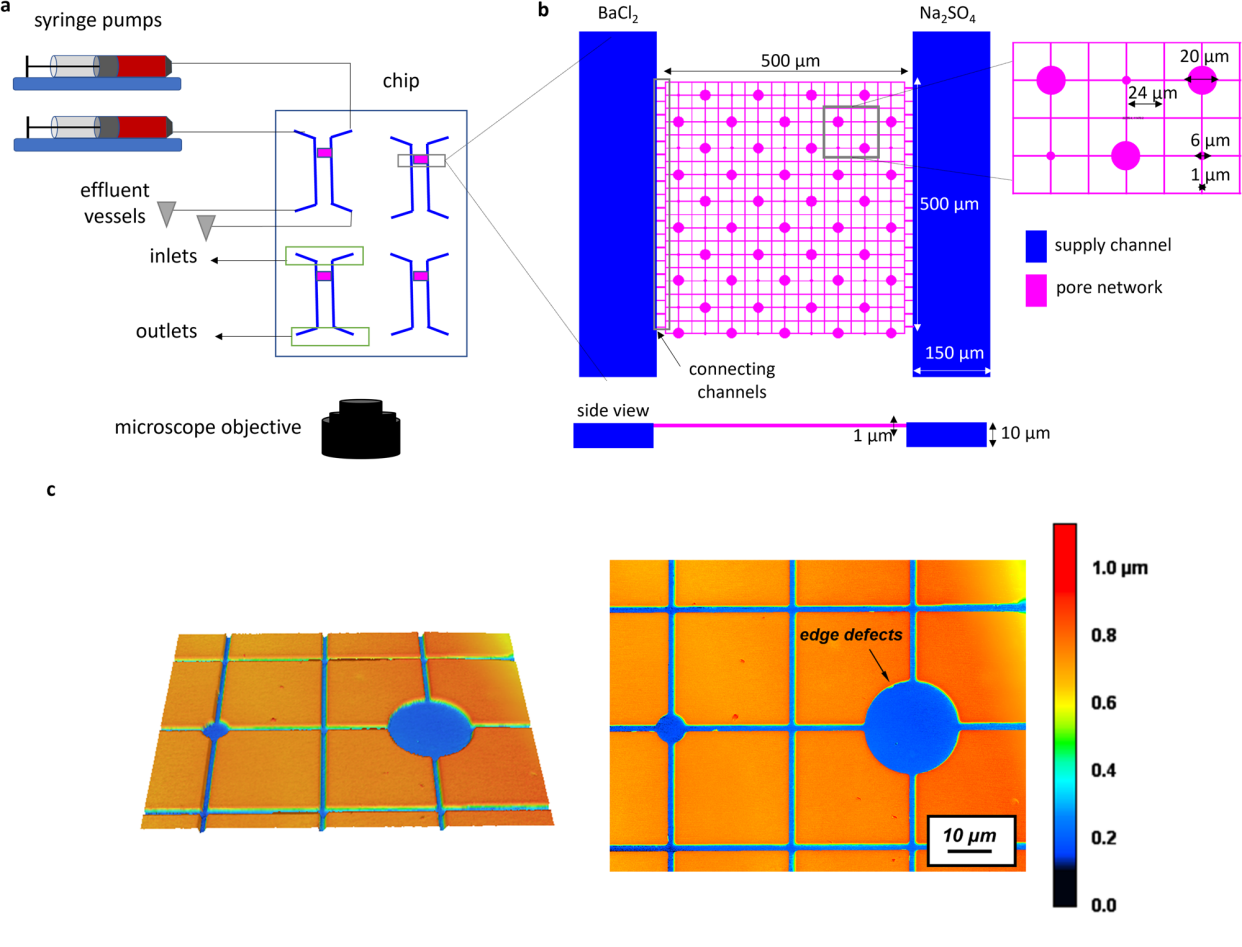
Experimental setup with chip design for the precipitation of barite in a microporous medium. **a** Schematic representation of the microfluidic setup for transport-induced crystallization in a pore network with a microfluidic chip connected to syringe pumps and effluent vessels; **b** enlargement of top and side view of the pore network connected to the supply channels; **c** laser confocal microscopy image of the pore network showing its depth with the color scale marking 0 and 1 µm the bottom surface and the top surface, respectively.

**Fig. 5:**
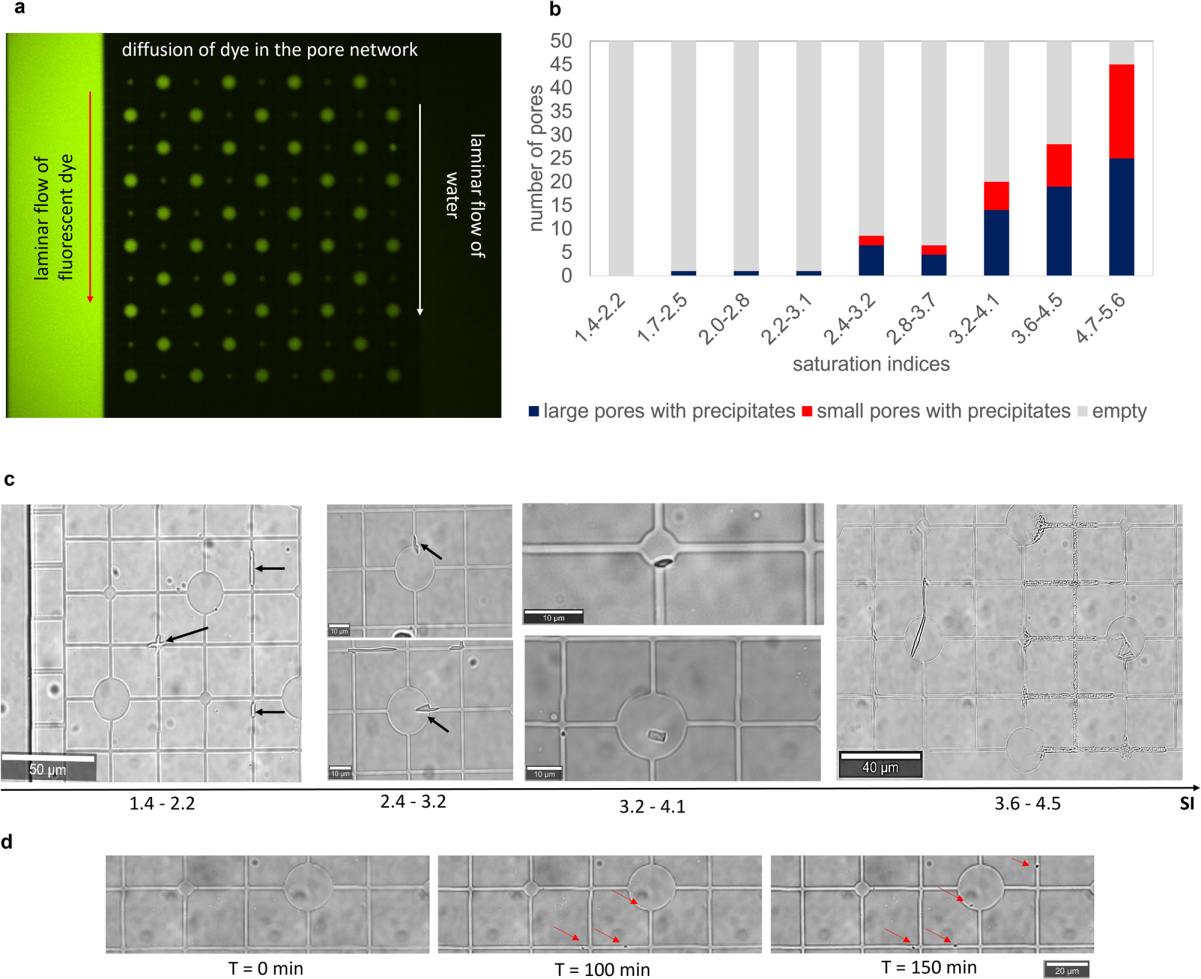
Mineralization of barite in the pore network. **a** Tracer test (fluorescent dye) conducted before experiments to illustrate the diffusion of solutes across the pore network with a decrease in intensity from left to right; **b** number of large and small pores containing precipitates after 3 h in experiments with different SI ranges; **c** micrographs representative of the experiments showing the trends in the location of precipitates as a function of the saturation index (SI); **d** micrographs of temporal evolution at a selected location from an experiment with 2.4 < SI < 3.2 with arrows indicating the formation of crystallites on the surface of the PDMS.

In the present study the level of complexity was increased by coupling nucleation with mass transport in a medium of variable pore sizes within the same experiment. The goal was to investigate whether the inclusion of a transport component mitigates the effect of pore size dependency on nucleation dynamics. To this aim, we induced the precipitation of barite in a pore network consisting of large (20 µm diameter) and smaller (6 µm diameter) cylindrical pores interconnected by squared capillaries of 1 µm^2^ squared cross-sectional area. This pore network (cf. methods section) is connected to adjacent supply channels, where reacting solutions were injected (Fig. 4b). Several experiments with varying solution concentrations were conducted. The injection of reacting solutions at a constant rate in the supply channels generates a diffusive solute flux from the supply channels into the pore network as demonstrated by the fluorescein tracer test conducted prior to the experiments (Fig. 5a). When Ba and sulfate solutions meet toward the center of the chip due to counter-diffusion, the solution becomes oversaturated and eventually barite precipitates as a given supersaturation threshold is exceeded.

The fluxes and the concentrations of the reacting solutes in the pore network were calculated using COMSOL Multiphysics, based on the exact pore network geometry. This allowed to evaluate the solute species concentrations at every point in space and time in the pore network. By coupling this information with geochemical speciation calculations using GEMS^43^ (see Table S2 and Fig. S4 in Supplementary Note 4) the corresponding SIs with respect to barite were determined. The frequency of occurrence of crystallites in small and large pores after 3 h is shown in Fig. 5b. The micrographs monitoring nucleation events in the pore network are shown in Fig. 5c as a function of increasing SI (i.e., from low to high concentration experiments). At SI < 2.2 nucleation occurred only in the capillaries (Fig. 5c, SI = 1.4–2.2), while at higher SI (SI 2.4–3.2; Fig. 5c), barite precipitation started in the capillaries and later extended into the larger pores, where solutes were available for further crystal growth. At SI > 2.5 nucleation of barite was observed in the pores, with crystallites appearing preferentially in the larger pores (Fig. 5c; SI 3.2–4.1). In general, preferential barite precipitation was observed in the larger pores (for 2.4 < SI < 4.5; see Fig. 5b, c). At high SI (i.e., SI > 4.5), the pore size effect seems to vanish, there is no preferential nucleation in small or large pores. In these experiments, the pore network was clogged within 30 minutes, blocking the connection between the supply channels. The nucleation started most of the time along the surfaces of the pore network at the corners of the polydimethylsiloxane (PDMS; see red arrows in Fig. 5d) which act like cracks and pits, possibly constituting energetically favorable nucleation sites since they maximize the contact area between the substrate and the precipitating crystals^44^.

**Fig. 6:**
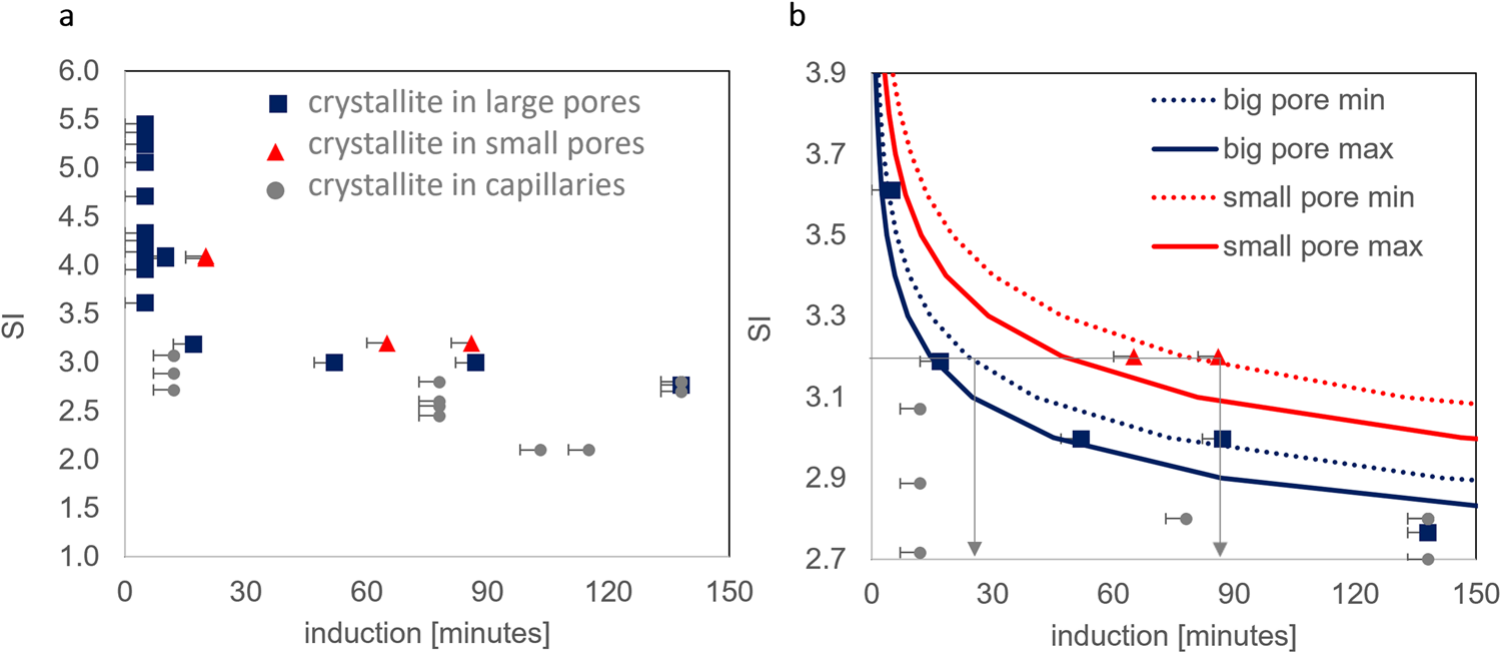
Supersaturation-nucleation diagram. **a** Calculated SI versus the measured induction time for nucleation in the large and small circular pores and in capillaries; **b** enlargement of graph (**a**) for the SI region between 2.7 and 3.9 with additional theoretical CNT calculations (based on Eq. 5) showing maximum (max) and minimum (min) induction times based on the maximum and minimum of the experimentally determined nucleation rates. The arrows in (**a**) indicate the increased induction time for large and small pores at SI = 3.2. N.B. several points in (**a** and **b**) are superimposed. The associated error for the induction time is given as the time interval for each picture.

The solute concentration at any location in the pore network is mainly controlled by the distance to the supply channels. To decouple the effect of precipitation on aqueous solution concentration (local depletion of Ba and SO_4_^2−^) in the nucleation-supersaturation diagram^31^ (Fig. 6a, b), only the induction time of the first crystallite per horizontal channel (i.e., the channel connecting the two supply channels) was measured. The nucleation-supersaturation diagram^31^, relates the SIs to the measured induction time. It correctly predicts that at high SIs (SI > 4), there should be no significant difference in induction times for large and small pores. For SI between 3 and 4, the CNT calculations also agree with the experimental data, as they correctly predict that the induction times are up to 50–70 min shorter for the larger pores compared to the smaller pores. For SI < 3, crystallites were observed mostly in the capillaries, usually with smaller induction times than in the pores. Additional Raman measurements conducted on precipitates in the capillaries confirmed that the crystallites consisted of pure crystalline barite (no amorphous phases).

The theoretical induction time based on CNT was calculated using Eq. 38 from Kashchiev and Van Rosmalen^45^:

6 $${{t}_{{{{{{\rm{i}}}}}}}=\left(\frac{3{\alpha }_{{{{{{\rm{v}}}}}}}}{\pi {A}^{3}J}\right)}^{\frac{1}{4}}$$

where *α*_v_ is the detectable volume fraction of the new precipitate (usually based on an optical resolution of 500 nm^2^ per pixel) to the pore volume, $A=\frac{{{{{{{\rm{d}}}}}}R}}{{{{{{{\rm{d}}}}}}t}}$ is the growth rate of a crystallite with radius *R* (evaluated at 6.67 × 10^-9 ^m s^−1^ for equimolar reacting solutions of 2.5 mM) and *J* is the nucleation rate as determined from the optical images. The parameter *α*_v_ is determined as the ratio of the detectable volume *V*_d_ of the nucleating phase to the volume of the pore, *V*_p_, yielding ${\alpha }_{{{{{{\rm{v}}}}}}}=\frac{{V}_{{{{{{\rm{d}}}}}}}}{{V}_{{{{{{\rm{p}}}}}}}}$, with *V*_d_ assumed to be 1 µm^3^, corresponding approximately to the resolution of the optical microscope.

The nucleation rate range, *J*, of barite as a function of supersaturation was calculated using the equations summarized in Supplementary Note 4 and inserted into Eq. 6 to determine induction times. For the sake of clarity, only the theoretically calculated induction times for equimolar concentrations of 2.5 mM (2.8 < SI < 3.2) are plotted in Fig. 6b. The concentration gradient of BaSO_4_ monomers in the pore network results in a window for the nucleation rate with a *J*_min_ and *J*_max_ and consequently a range for the induction time as plotted in Fig. 6b. The theoretical saturation-nucleation diagram shows, as expected, that for a given SI, the induction time increases with decreasing pore volume.

## Discussion

### Kinetics versus thermodynamic control on nucleation in confinement

The experiments conducted with droplets capture the nucleation of barite in well-defined volumes of aqueous solution. The analysis shows that the probability of nucleation of barite scales with the volume of solution as commonly reported also for protein crystallization^24^ and the precipitation of carbonates^46^. The reason for the retardation of nucleation events in the nL volumes of the droplets compared with the bulk solution experiment is due to the much smaller availability of surfaces that can act as favorable substrates for heterogeneous nucleation in the droplet experiments. In the bulk solution experiment the glass slide or impurity particles—typically present between 10^6^ and 10^8^ per mL^24^ in bulk solutions—may boost nucleation. Although the reacting solutions were filtered (c.f. “Methods” section) in the droplet experiments, fine impurity particles cannot be completely removed from the solution. However, such particles are distributed into several hundreds of generated droplets, decreasing the probability of heterogeneous nucleation in each droplet.

Equation 4 can be rewritten in logarithmic form as Eq. 7, with the induction time, *t*_i_, as a function of the pore volume:

7 $${{{{\mathrm{lg}}}}}{t}_{i}={{{{\mathrm{lg}}}}}\, {{{{\mathrm{ln}}}}}\,{P}_{0}^{-1}-{{{{\mathrm{lg}}}}}J-{{{{\mathrm{lg}}}}}V$$

**Fig. 7:**
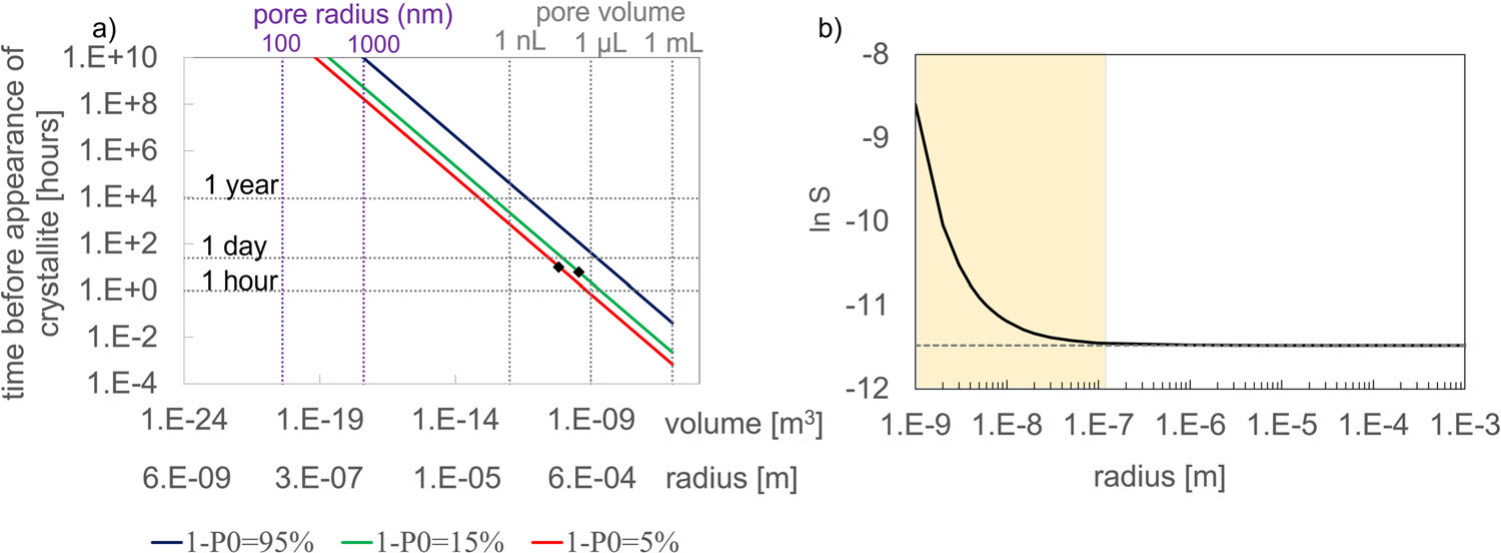
Kinetic versus thermodynamic retardation of barite precipitation as a function of pore size. **a** Probability of nucleation based on experiments B and C giving the induction time with the pore volume in [m^3^]; to ease the interpretation, the pore volume and radius in more conventional units are given by the axes on top of the graph; **b** change of the effective solubility of BaSO_4_ with pore radius. The shaded region shows the region where PCS is effective.

Figure 7a depicts the induction time calculated by Eq. 7 as a function of the pore volume; it shows specified probabilities (5%, 15% and 100%) of nucleation as a function of the droplet volume. The probability of 95% nucleation increases from 1 day to 1 year as the volume decreases from 1 µL to 1 nL. Similarly, in a pore with a radius of 1000 nm or 1 µm (volume 4.19 × 10^−18^ m^3^), barite nucleation is unlikely to occur (induction time >1 million years). Moreover, the effective solubility of barite as a function of pore radius plotted in Fig. 7b (see Supplementary Note 1 for equation) indicates that the precipitation of barite from a supersaturated solution can also be thermodynamically hindered in nano-porous media due to pore-size controlled solubility. From our analysis, we conclude that the retardation of barite nucleation is operating in pores with a radius of less than 1 µm while the thermodynamic (PCS) effect would only be significant for a pore size of less than 100 nm. Therefore, for the present experiments, contrary to nanoporous media such as silica gels or clays, the latter effect can be excluded, simplifying the analysis of the results.

### Nucleation in micro-porous media and the role of transport

In the pore network experiment, a substrate is available for heterogeneous nucleation to occur and there is a continuous influx of reacting solutes. Consequently, the induction time is much shorter than the estimated induction time for a droplet of 1 µm radius with a finite reservoir of solutes. In general, even in the pore network, barite nucleates faster in the larger pores. The experimental induction times are within the range of the theoretical induction times based on CNT.

At lower SIs, unexpected barite precipitation was observed in the capillaries with a shorter induction time than in the pores; these were the only locations where barite precipitated at SI < 2.2. Nucleation results from a competition between the volume (*V*) versus the surface area (SA) of the nucleus and should thus depend on the local SA/V ratio (cf. Eq. 1). The capillaries exhibit a larger surface area per unit volume of fluid (3.0 × 10^6^ m^2^ m^−3^) compared to the large pores (1.2 × 10^6^ m^2^ m^−3^), with an increase in pitches defects at the corners (see pre-characterization of surface topography of the pore network in Fig. 4c), thus increasing the number of nucleation sites and consequently the nucleation rates. This phenomenon was also observed in nanopores, where functionalization of the surface increased the number of nucleation sites, counteracting the generally observed “inhibition of nucleation in nanopores”^29^. Furthermore, kinetic Monte Carlo simulations have also demonstrated that the surface roughness of a solid substrate enhances the local concentration of clusters near the surface of the substrate. This usually increases the heterogeneous nucleation rate^47^. The fact that the surface topography and irregularities in the capillaries become significant at low SI maybe be due to a combination of the increase in the size of the critical nucleus required for nucleation to occur with decreasing SI^45^ and the proximity effect^32,33^ generated by the surface irregularities.

Already in the small spatial and temporal scale experiments, the effect of different pore size distributions and shapes leads to heterogeneous mineralization. In the subsurface when solute transport is controlled by diffusion (e.g., in clays) such behavior can be more pronounced. Depending on the nature of the porous medium and the spatial distribution of pores with different sizes, heterogeneous precipitation patterns may develop with small pores remaining open and connected, while larger ones are clogged, which in consequence could also lead to heterogeneous flow fields in the subsurface. Thus, besides the PCS effect^48,49^, nucleation kinetics^50,51^ with the inclusion of the pore size dependence and consideration of nucleation sites should also be implemented in reactive transport models to predict crystallization processes in porous media and their consequences on subsurface evolution. Future work will numerically assess the effects of barite crystallization in rock matrices with a heterogeneous pore size distribution and their consequential changes on transport properties.

## Methods

### Pseudo batch experiment

The nucleation pathway of barite in bulk solution was investigated. A solution of 0.75 mM of BaCl_2_ and a solution of 0.75 mM of Na_2_SO_4_ was prepared and filtered using MF 187MilliporeTM Membrane Filters of 0.45 μm pore size. 10 µL of the Na_2_SO_4_ solution followed by 10 µL of the BaCl_2_ solution were pipetted in a petri dish with a 0.17 µm glass base (ref 150680 from Thermo Scientific). The mixed solution in the petri dish was continuously analyzed by Raman spectroscopy using a WITec alpha300 Ri Inverted Confocal Raman Microscope, WITec Wissenschaftliche Instrumente und Technologie GmbH, Ulm, Germany) with a ×50 objective (numerical aperture NA 0.8; Nikon, Tokyo; Fig. 1a). The instrument is equipped with a 70 mW Nd: YAG laser (*λ* = 532 nm) and a thermoelectrically cooled charge-coupled device (CCD). The laser power was set to 30 mW with a grating of 600 mm^−1^ over a spectral range of 0–4000 cm^−1^ was selected. The measuring per spectral was set to 10 s (20 measurements each of 0.5 s). Before the addition of BaCl_2_, the Raman spectrum of the Na_2_SO_4_ solution was also collected. This experiment was repeated by time-lapse optical microscopy using an inverted Microscope Eclipse Ti2 (NIKON, Tokyo) with a CFI Plan Fluor DL 10X objective (NA 0.3; W.D. 0.16, Nikon, Tokyo). This experiment, based on classical laboratory tools is an established methodology proposed by Montes-Hernandez and Renard^52,53^ to unravel crystallization pathways and has indicated prenucleation clusters for the case of calcite and siderite precipitation.

### Nano volume droplet generation

To investigate nucleation kinetics in a nanoconfined volume of water, a droplet generator microfluidic chip was used. Such systems have been used e.g., in geoscience to investigate the nucleation of calcium carbonate phases in confinement^41,46,54,55^. The reactors used in our experiments are based on the flow-focusing principle^56,57^ to generate droplets, whereby a carrier phase (oil) pinches off a dispersed phase (aqueous solution) into droplets as it passes through a nozzle (see Fig. 2). The size of the droplets is controlled by the flow rate of the continuous carrier phase, the nature of the dispersed phase and the nozzle size.

To generate droplets of different sizes, 3 chip designs labeled A–C (Fig. 2) made from a rigid transparent polymer (Topas COC) were used. Experimental setups A and B consisted of a droplet generator chip connected to a storage chip (made of polycarbonate), where the droplets were monitored after fabrication. The experimental setup C consisted of a droplet generator and an integrated storage platform for monitoring. The principle of droplet generation is the same for all the experiments. Two inlets were dedicated to the injection of the oil phase (Novec™ 7500 fluorinated oil, Fluigent Le Kremlin-Bicetre, France), and two for the injection of the dispersed (aqueous) phase, which was a mixture of 0.75 mM of Na_2_SO_4_ and 0.75 mM of BaCl_2_. The flow rates used for each experiment are indicated in Fig. 2. The size of the droplets generated using this technique is given in Fig. 2e; the volumes were calculated using Eq. 16 in dos Santos et al.^58^. (see documentation and Fig. S5 and Table S2 in Supplementary Note 5). The frequency of nucleation in the droplets was monitored by time-lapse optical microscopy using an inverted Microscope Eclipse Ti2 (NIKON, Tokyo) with a CFI Plan Fluor DL ×10 objective (NA 0.3; W.D. 0.16, Nikon, Tokyo) and a high-resolution camera from Zyla (sCMOS, Andor, Belfast) in DIC mode for 6–10 h. The images collected were analyzed using an image processing methodology detailed in Supplementary Note 6 (Figs. S6 and S7), to output the frequency of nucleation with time.

### Automated image analysis of droplet experiments

A python-based automated image analysis procedure for the quantification of barite nucleation and evolution in the storage chip was proposed for evaluating the optical microscopy micrographs from the experiments. Based on the clarity of the micrographs, certain regions were cropped and used for further analysis. This decision was validated by comparing the ratio of droplets where crystals had formed (*N*_0_) to the total number of droplets (*N*) in the selected cropped regions with the ratio found in a much bigger portion of the image at the initial state. The micrographs were then preprocessed and prepared for analysis by denoising them using a non-local mean denoising algorithm^59^. The micrographs were binarized using an adaptive thresholding technique^60^ and furthermore treated using two morphological operations, the dilation and erosion operations, to ensure that the droplets were completely enclosed and were not affected by the previous preprocessing steps. Further image analysis procedures were then applied to identify the droplets and to remove the outliers that may have resulted from instabilities in the flow (giving rise to slightly larger or smaller droplets). The next step in the image analysis process was to identify the nucleation of barite throughout all the experiments.

### Transport-induced crystallization in a pore network

The microfluidic reactor used here is composed of two adjacent supply channels of 150 µm width and a 500 µm × 500 µm pore network (Fig. 4). The narrow connecting channels of 2 µm width between the supply channels and the pore network enables a diffusion-dominated transport regime in the pore network. The pore network consisted of large circular cylindrical pores (i.e., not spheres) of 20 µm diameter and smaller cylindrical pores of 6 µm diameter interconnected by squared capillaries of a 1 µm^2^ squared cross-sectional area). The depth of the pore network was 1 µm, while the supply channels had a depth of 10 µm. The microfluidic reactor was made from PDMS and closed with a glass cover following the procedure detailed in Poonoosamy et al.^61^. The surface topography was analyzed using confocal laser microscopy (VK-9710 K, Keyence, Japan) showing an increase in surface heterogeneity along the edges of the capillaries and pores. These surface irregularities act as nucleation sites. The two inlets were each connected to a 2.5 mL glass syringe and the two outlets were linked to two effluent vessels. The microfluidic reactor was initially filled with deionized water, followed by the injection of the reacting solutions (BaCl_2_(aq) and Na_2_SO_4_(aq)) at a rate of 800 nL min^−1^ using a syringe pump (Nemesys, Cetoni GmBH, Germany) for 48 h. The diffusion of the reacting solutions fostered the precipitation of BaSO_4_ in the pore network of the microfluidic device, which was monitored by optical microscopy (WITec alpha300 Ri Inverted Confocal Raman Microscope) with a ×100 objective. The experiments were conducted at ambient temperature (21 °C) and pressure with equimolar concentrations of BaCl_2_ (aq) and Na_2_SO_4_ (aq). The concentrations of the reacting solutions were varied (0.325, 0.5, 0.75, 1, 1.25, 2.5, 5, 10, 100 mM) to determine the dependence of the induction times on the SI. Each experiment was duplicated. The initial SIs were calculated following the steps described in Poonoosamy et al. ^35,61^. which consisted of an initial evaluation of the flow and concentration field using COMSOL Multiphysics, and secondly the geochemical calculations of the aqueous speciation for the determination of the SI using GEMS selector^43^. The range of SI associated with the reacting solutions is given in Table S2 in Supplementary Note 4. To decouple the effect of transport on the precipitation mechanism and vice-versa, only the time of appearance of the first crystallite in each horizontal channel (connecting the supply channels) as well as the number of large and small pores filled with BaSO_4_ after 3 h were recorded. N.B.: Before any experiment, the pore network was flushed for 24 h with filtered Mili Q water to ensure that the surface properties of the PDMS were the same for all experiments as the wetting properties of PDMS are altered by water.

### Supplementary information

Supplementary Information

## Data Availability

Data generated and analyzed supporting Figs. 1–3 and 5 are publicly available in the Jülich Data repository (10.26165/JUELICH-DATA/2SCFCA). Any other datasets generated (images or simulation file) during the current study are also available from the corresponding author upon reasonable request.

## Code availability

No custom code nor mathematical algorithm that is central to the conclusions of this work was developed. Python scripts for processing images are also available from the corresponding author upon reasonable request.
