# Supplementary material for: Microfluidic investigation of pore-size dependency of barite nucleation
Source: Commun Chem. 2023 Nov 16;6:250. doi: 10.1038/s42004-023-01049-3 (PMC10654916; doi:10.1038/s42004-023-01049-3)
Supplement: Supplementary file 1 — Supplementary Information [file 42004_2023_1049_MOESM1_ESM.pdf]

Supplementary Information

MICROFLUIDIC INVESTIGATION OF PORE SIZE DEPENDENCY OF BARITE NUCLEATION

Jenna Poonoosamy[1], Abdulmonem Obaied[1], Guido Deissmann[1], Nikolaos I. Prasianakis[2], Moritz Kindelmann[3], Bastian Wollenhaupt[4], Dirk Bosbach[1], Enzo Curti[2]

1 Institute of Energy and Climate Research (IEK-6): Nuclear Waste Management, Forschungszentrum Jülich GmbH, 52425 Jülich, Germany

2 Laboratory for Waste Management, Paul Scherrer Institut, CH-5232 Villigen PSI, Switzerland

3 Ernst Ruska-Centre for Microscopy and Spectroscopy with Electrons (ER-C 2): Materials Science and Technology, Forschungszentrum Jülich GmbH, 52425 Jülich, Germany

4 Institute of Bio- and Geosciences (IBG-1): Biotechnology, Forschungszentrum Jülich GmbH, 52425 Jülich, Germany

Corresponding author: Jenna Poonoosamy

email: j.poonoosamy@fz-juelich.de

Supplementary Note 1

Equation for pore size-controlled solubility (PCS)

When a crystal grows from a solution in a pore of a porous material, its surface tension will give rise to excess pressure p within the crystal[1]. For cylindrical pores with radius *r* (**Fig. S1.1**) this excess pressure can be approximated by

$$p = \frac{-2\gamma cos\theta}{r} \qquad \text{(S1.1)}$$

where the factor of 2 results from the cylindrical pore geometry and γ is the surface tension [N m^{-1}] and θ is the contact angle between the pore wall, crystal, and liquid interface. The pressure increases for decreasing pore sizes.

The excess pressure p in a crystal can shift the solubility S of the solid from the bulk solubility S_0 and is given as[2,3]:

$$p = (n^+ + n^-)RT\left[\frac{1}{v_S^c}\, ln\left(\frac{S}{S_0}\right) - \frac{Xv_w^l}{v_w^c}(S - S_0)\right] \qquad \text{(S1.2)}$$

where n$^+$ and n$^-$ are the numbers of positive and negative ions the solid dissociates into, R is the universal gas constant [J mol^{-1} K^{-1}], T is the temperature [K], v_w^c the molar volume of the ssolid phase (m^3 mol^{-1}), v_w^l the molar volume of water in the liquid phase [L mol^{-1}], and v_w^c the molar volume of the water in the solid [L mol^{-1}]. The factor X (=1000) accounts for the conversion of mol L^{-1} to mol m^{-3}. The ratio of v_w^l and v_w^c accounts for the hydration of the solid phase. In the case of anhydrous crystals like barite the equation is written as:

$$p = nRT\frac{1}{v_S^c}\, ln\left(\frac{S}{S_0}\right) \qquad \text{(S1.3)}$$

The solubility of a salt inside the pores can be different from that in a bulk solution and increases when the stress on the crystal is compressive. Combining equations S1.1 and S1.2, the solubility can be written as a function of the pore radius[4]:

$$ln\frac{S}{S_0} = \frac{2\gamma v_S^c}{(n^+ + n^-)RTr} \qquad \text{(S1.4)}$$

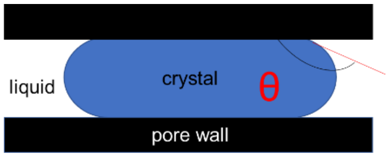

Figure S1 Crystal growing in a cylindrical pore.

N.B.: In the case of barite, the solubility, S_0, is the square root of the solubility product constant K_{sp} (cf. Supplementary Note 3).

Supplementary Note 2

Temporal evolution of bulk experiment and associated Raman measurements

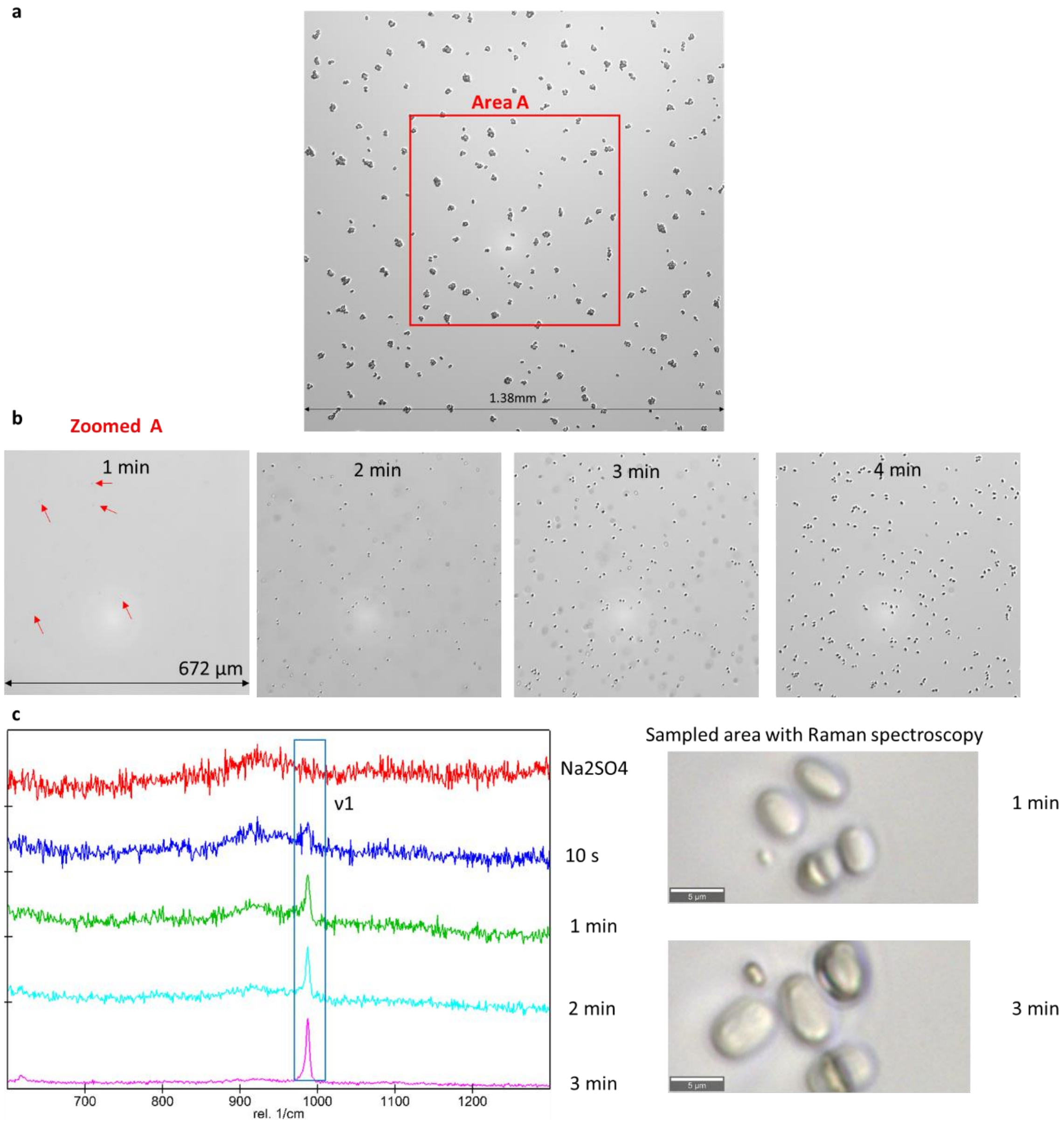

Figure S2 a Micrograph of bulk solution in petri-dish after 15 minutes, with **b** a zoom in for the first 4 minutes (with red arrows indicating first visible crystallites), and **c** associated Raman spectra with a measuring time of 50 s (0.5 ms x100 integration and x100 objective).

These additional measurements are in agreement with the results reported in the main document with a much shorter measuring time of 5 s.

Supplementary Note 3

Calculation of nucleation rates of barite based on CNT

The nucleation rate depends on supersaturation and can be calculated as follows

$$J = \mathrm{r}exp\left(-\frac{\Delta G_c}{kT}\right) \qquad \text{(S3.1)}$$

where k is the Boltzmann constant, T is the absolute temperature (298.15K), r a pre-exponential factor and ΔG_c is the energy required for the formation of a nucleus of critical size. ΔG_c is given as:

$$\Delta G_c = \frac{\beta {v_0}^2 \sigma^3}{(kTln\Omega)^2} \qquad \text{(S3.2)}$$

where v_0 is the molecular volume of the solid phase (8.6×10^{-29} m^3 for barite), β is a geometry factor that depends on the shape of the nucleus and was set to 16.8 (value for spheres according to Nielsen[5]), and σ (J m^{-2}) is the specific surface energy of the cluster/solution interface set to 0.134 Jm^{-2} for barite[6]. Ω is the saturation ratio given as $\Omega = \frac{\{Ba^{2+}\}\{SO_4^{2-}\}}{K_{sp}^0}$, with the saturation index SI defined as $SI = \log_{10}\Omega$.

The pre-exponential factor, *r*, includes a volume diffusion step and is given as:

$$\mathrm{r} = 2\pi ZDN_0N_1d_c \qquad \text{(S3.3)}$$

where D is the diffusion coefficient of $BaSO_4$ monomers in free water[7] set to 9.3×10^{-10} m^2 s^{-1} and $d_c = \frac{4\sigma v}{kTln\Omega}$. N_1 and N_0 are concentrations that represent the number of monomers per unit volume of fluid and the number of nucleation sites, respectively.

The values for N_0 are usually fitted values. For the interpretation of our results, we used the values reported for barite in Prieto[7], given in Table S3 as case 0.

N_1 depends on the solute concentrations in the aqueous solution and refers to the amount of aqueous $BaSO_4$ molecules per m^3; their concentration was determined from speciation calculations using GEMS[8].

Z is the Zeldovich factor given as:

$$Z = \sqrt{\left(\frac{\Delta G_c}{3\pi kT(n_c)^2}\right)} \qquad \text{(S3.4)}$$

with the number of monomers in the critical nucleus, n_c, given as:

$$n_c = \left(\frac{2\sigma a}{3kTln\Omega}\right)^3 \qquad \text{(S3.5)}$$

where $a = 9.42\times10^{-19}$ m^2 is the area occupied by a molecule of $BaSO_4$[7].

In the case of 3D nucleation, σ is replaced by an effective specific surface energy σ_{eff} (J m^{-2}) given as:

$$\gamma_{ef} = \Psi^{\frac{1}{3}}(\theta)\gamma \qquad \text{(S3.6)}$$

where $\Psi(\Theta) = (\frac{1}{4})(2 + cos\Theta)(1 - cos\Theta)^2$. n_c and the pre-exponential factor, *8*, were re-evaluated accordingly.

We used the values for N_0 as reported by Prieto[7] for calculating the nucleation rate for the section "Barite nucleation in confinement" i.e. (droplet experiment). In addition to values reported by Prieto[7], we conducted a sensitivity analysis (case 1 and 2) to quantify the impact of N_0 (HET) on the nucleation rate using different contact angles: 30° for hydrophilic surfaces, 80° for hydrophobic surfaces, and 72.9° as a fitted value to match our experimentally measured rates in the droplets. As reported in the following Table S3, the nucleation rate scales proportionally with the values of nucleation sites (N_0). This follows directly from eq. S3.1 and S3.3. In contrast, the nucleation rate (J) has an exponential dependence on the contact angle, implying that small changes in the contact angle change the nucleation rate (J) by several orders of magnitude. These effects including (i) HOM proceeds at a much faster rate than HET (ii) the influence of N0 on the computed nucleation rate and (iii) the influence of θ on the nucleation rate, are also captured by Figure S3 which depicts the nucleation rate as function of SI for various calculation cases presented in Table S3. Based on the data reported in Table S3, in our experiments heterogeneous nucleation is most likely involved; homogeneous nucleation can be excluded based on the vanishingly small, calculated values of J compared to the experimentally observed rates.

Table S1 Influence of N_0 and contact angle (θ) on the nucleation rate calculated for an SI of 2.8 using a fixed N_1 of 3.65×10^{22} m^{-3}. (N.B the highlighted row indicates the contact angle value fitted to the presented experimental data).

Calculation case	N_0 [m^{-3}]	θ [°]	J [$m^{-3}s^{-1}$]
Homogeneous nucleation			
Case 0a (N_0 based on Prieto)	$3.3\cdot10^{28}$	n.a.	$6.69 \times\cdot10^{-14}$
Case 1a	$3.3\cdot10^{27}$	n.a.	$6.69\times\cdot10^{-15}$
Case 2a	$3.3\cdot10^{29}$	n.a.	$6.69 \times\cdot10^{-13}$
Heterogeneous nucleation			
Case 0b (N_0 based on Prieto)	$2.5\cdot10^{13}$	30	$6.52 \times\cdot10^{15}$
Case 1b	$2.5\cdot10^{12}$	30	$6.52 \times\cdot10^{14}$
Case 2b	$2.5\cdot10^{14}$	30	$6.52 \times\cdot10^{16}$
Case 0c (N_0 based on Prieto)	$2.5\cdot10^{13}$	80	$2.01 \times\cdot10^{4}$
Case 1c	$2.5\cdot10^{12}$	80	$2.01 \times\cdot10^{3}$
Case 2c	$2.5\cdot10^{14}$	80	$2.01 \times\cdot10^{5}$
Case 0d (N_0 based on Prieto)	$2.5\cdot10^{13}$	72.9	$2.84 \times\cdot10^{0}$
Case 1d	$2.5\cdot10^{12}$	72.9	$2.84 \times\cdot10^{-1}$
Case 2d	$2.5\cdot10^{14}$	72.9	$2.84 \times\cdot10^{1}$
Experimental results	n.a.	n.a.	$2.0 \times\cdot10^{4}$-$2.0 \times\cdot10^{6}$

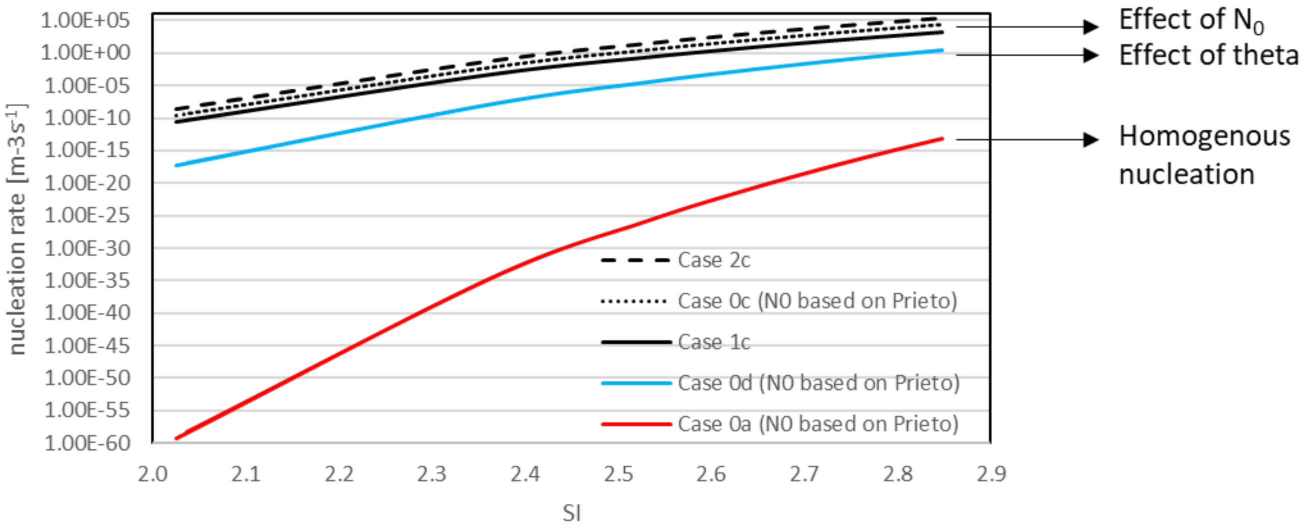

Figure S3 Nucleation rate of barite as function of saturation index (SI) for various combinations of N_0 and contact angle θ (cf. **Table S3**).

For the calculation of the nucleation rate and resulting induction time presented in **Figure 6a-b**, i.e., the pore network experiment, the number of nucleation sites was set to 2.5×10^{13} m^{-3} (i.e., the same value as in Prieto[7]) for the large pores. Since such a value depends on available surface area, decreasing the pore radius from 20 to 6 µm involves a reduction of the surface area by a factor of 8.5. N_0 for the small pores was thus estimated to decrease by the same factor, i.e., $N_0 = 2.9\times10^{12}$ m^{-3} for the small pores. For the 2.5 mM reacting solutions, the initial SI lies between 2.8 and 3.7 resulting in N_{1min} and N_{1max} of 2.11×10^{13} and 1.5×10^{13} monomers per m^3, respectively. The calculated J_{min} and J_{max} provide a range of nucleation rates for the large and small pores based on the maximum and minimum concentration of $BaSO_4$(aq) monomers. Nucleation starts on the surface of PDMS and not on the plasma-treated glass[8] and consequently the contact angle was set to 81° (based on previous work and within the range expected for PDMS[9]). Using these values, we calculated the apparent induction time (equation 6 in main document) for the 2.5 mM reacting solutions (2.8<SI<3.7) and compared the results with the corresponding experimental datasets.

Supplementary Note 4

Velocity fields and aqueous solute distributions in the pore network

The velocity fields and the distributions of solutes were simulated using Comsol Multiphysics at a transient state (i.e., the first 30 minutes of the experiment) and at steady state. The simulation results for the 100 mM experiment are depicted in **Figure S4a-c**. A concentration gradient builds up 30 s after the start of the injection of the reactants, with the highest concentrations at the respective supply channel, which decreases to zero in the opposite channel. The compositions of the aqueous solutions along L1 and L2 were exported, and the geochemistry (activities of solutes) was solved using GEMS selector[10] to determine the saturation indices (SI). The saturation indices (SI) along lines L1 and L2 for the highest and lowest concentrations of solutes used in the experiments are plotted in **Figure S4d-g**. These graphs show that there is a negligible difference between the SI calculated for 600 s (10 minutes) after the start of the experiments and the steady state. Consequently, for any nucleation event after 600 s, the calculated steady-state SI was used in our analysis. The ranges of SI at steady state for the various experiments are given in **Table S4**.

Table S2 Calculated SI ranges associated with experiments conducted at different concentrations of reactants.

Equimolar reacting solutions of Na_2SO_4 and $BaCl_2$ [mM]	Range of saturation index (SI) at steady state
0.325	1.4 - 2.2
0.5	1.7 - 2.5
0.75	2.0 - 2.8
1	2.2 - 3.1
1.25	2.4 - 3.2
2.5	2.8 - 3.7
5	3.2 - 4.1
10	3.6 - 4.5
100	4.7 - 5.6

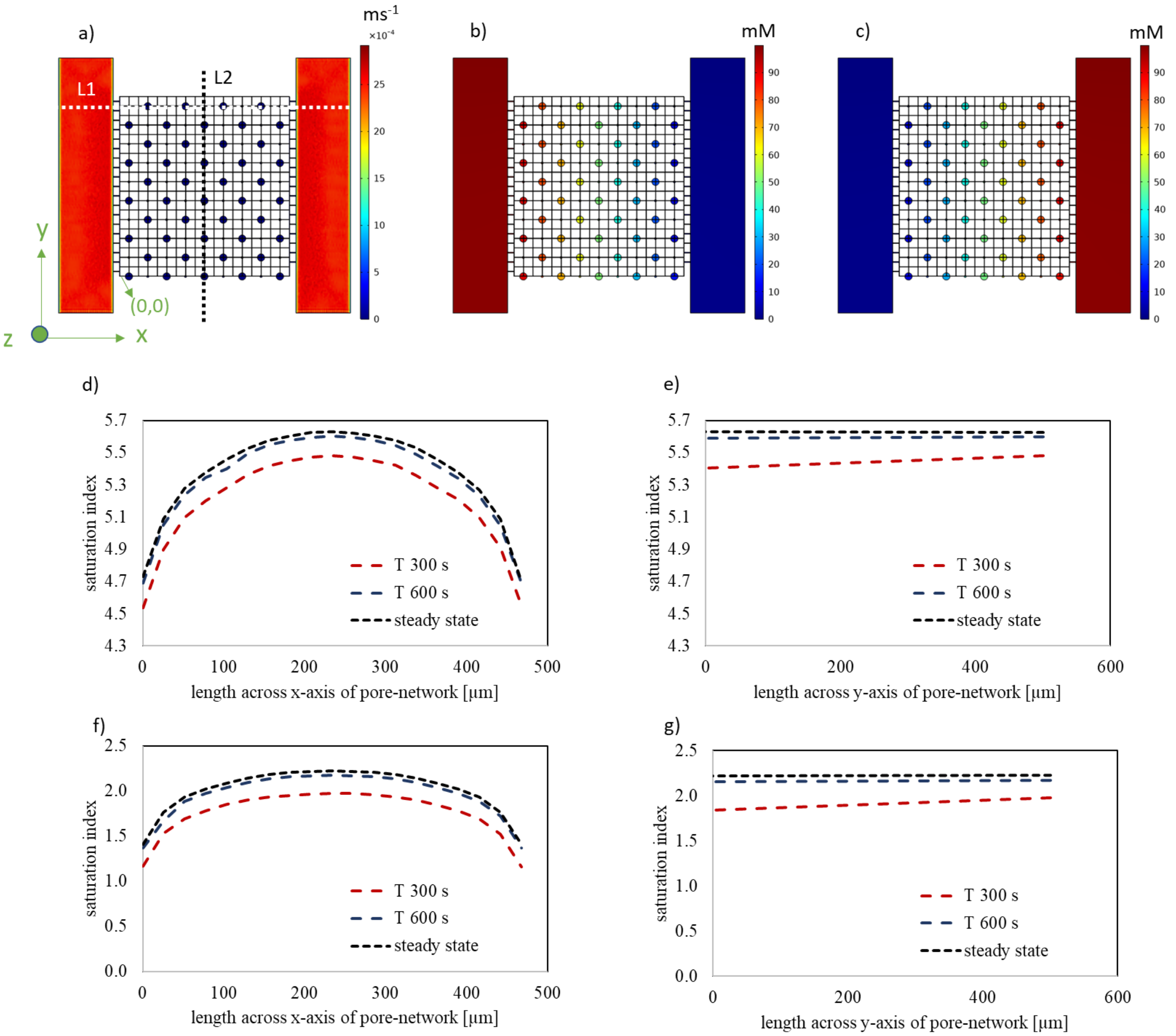

Figure S4 a Velocity field at steady state across plane Z=0.5 μm with numerical sampling lines L1 and L2, **b** and **c** simulated concentrations of Ba(aq) and SO_4(aq) across the pore network at steady state for the 100 mM solutions experiment (highest concentration injected); **d** and **e** temporal evolution of the saturation indices along lines L1 and L2, respectively, for the 100 mM solution experiment; **f**, and **g** temporal evolution of the saturation indices along lines L1 and L2, respectively, for the 0.325 mM solution experiment.

Supplementary Note 5

Determination of the volume of droplets

Droplets formed in microfluidic channels can either fill the cross-sectional area where they can be assumed to be rectangular cuboids or can be smaller than the channel depth and therefore assumed to be spheres. According to the images collected from the droplets in motion, they tend to adopt a cylindroid shape with rounded edges. The volume of these droplets can be calculated using the following equation given by dos Santos et al.[13]:

$$V = \frac{\pi}{4} hwL - \frac{\pi}{12} wh^2 \qquad \text{(S5.1)}$$

where h is the depth of the microfluidic channel, and w and L are the width and length of the cylindroid (**Figure S5**). The dimensions of the droplets and the calculated number of $BaSO_4$(aq) monomers (for reactant concentrations of 0.75 mM) for the different droplet microfluidic experiments are provided in **Table S5**.

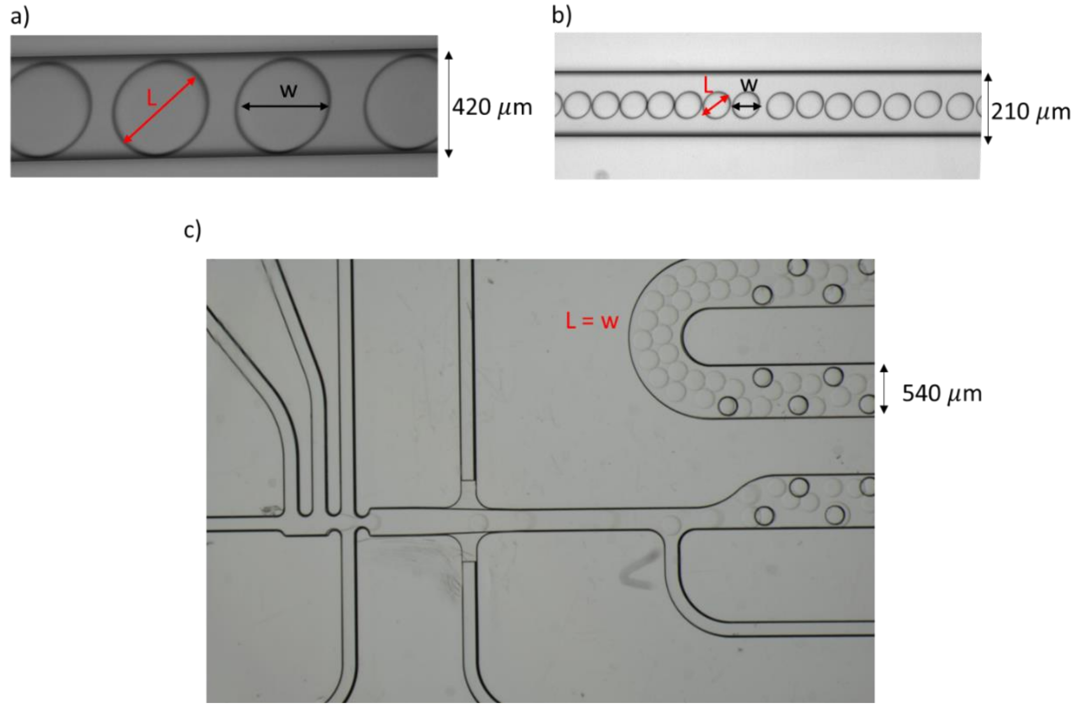

Figure S5 Illustration of the width and length of cylindroid shaped droplets; **a** for experiment A; **b** for experiment B; **c** for experiment C.

Table S3 Summary of size of droplets and calculated number of $BaSO_4$(aq) monomers (for reactant concentrations of 0.75 mM) for the different droplet microfluidic experiments A-C.

Design	L [µm]	W [µm]	h [µm]	Volume [nL]	Number of monomers
A	390	360	140	15	5.5×10^8
B	86	77	70	0.3	$1.1 \times\cdot 10^7$
C	173	173	100	1.9	$6.9 \times\cdot 10^7$

Supplementary Note 6

Image processing of droplet experiments

Given the increasing interest in studying nucleation, it is important to establish a robust and automated image analysis procedure to produce reliable and reproducible results that can be easily stored, analyzed, and compared with other experimental or simulated results.

Python packages

The analysis methodology was developed and implemented in the open-source Python programming language version 3.9.13[14], using the OpenCV-Python version 4.0.0.66[15], numpy version 1.21.5[16], and pandas version 1.4.4[17] libraries.

Datasets

Two datasets were analyzed: The first dataset comprised 20 images captured over 10 hours, with the first image taken 30 minutes after starting the experiment (experiment B). The second dataset comprised a single image taken 30 minutes after the start of experiment A. All the images had a size of 11934 x 6490 pixels. The original images were converted to 8-bit grayscale.

Cropping

For the first dataset, it was noticed that certain regions of the images could not be used because the storage chips (experiment B) started to crack after long exposure to fluorinated oil. For this reason, it was decided to crop two different regions from each image to be used for further analysis covering almost 212 droplets (see **Figure S6a**). To check the validity of this decision, the ratio of droplets containing crystals (N_0) to the total number of droplets (N) was compared between the selected cropped regions and a much bigger portion of the image, covering almost 550 droplets, at t=30 min (see **Figure S6b**). The results were found to be very compatible with the ratio at the selected cropped regions in **Figure S6a** being $\frac{N_0}{N} = 0.971$, and the ratio from the bigger cropped portion of the image (**Figure S6b**) being $\frac{N_0}{N} = 0.970$.

Preprocessing

The first step of preprocessing the cropped regions of the images was to treat the white noise, which, if left untreated, would make it difficult to identify small crystals in the droplets. The image denoising was performed using a non-local mean denoising algorithm[18]. This method proved to be effective in treating the white noise in the images as it scans different portions of the image to search for a pixel that resembles the pixel it wants to denoise. Additionally, to correctly identify existing droplets and the crystals formed inside them, it is necessary to convert the grayscale images into binary images. It is important to mention that applying conventional thresholding techniques, like Otsu's thresholding method, which uses a global thresholding value proved ineffective when treating the images in this case, due to existing variations in lighting throughout the analyzed regions in the images. For this reason, an adaptive thresholding technique[19] was utilized as it can analyze a small set of neighboring pixels at a time and can compute the threshold value for that specific local region before performing the binarization. Furthermore, two additional

morphological operations were performed on the binarized images to ensure that the droplet perimeters were completely enclosed to allow for accurate analysis of the images. With the use of a structuring element, a 3x3 kernel in this case, each morphological operation can identify the region to be examined around each pixel in the image. The dilation and erosion operations were applied consecutively to connect any parts of the droplet's outer perimeters that might have been removed by the previous preprocessing steps. These two morphological preprocessing operations were applied using the OpenCV-Python library[15].

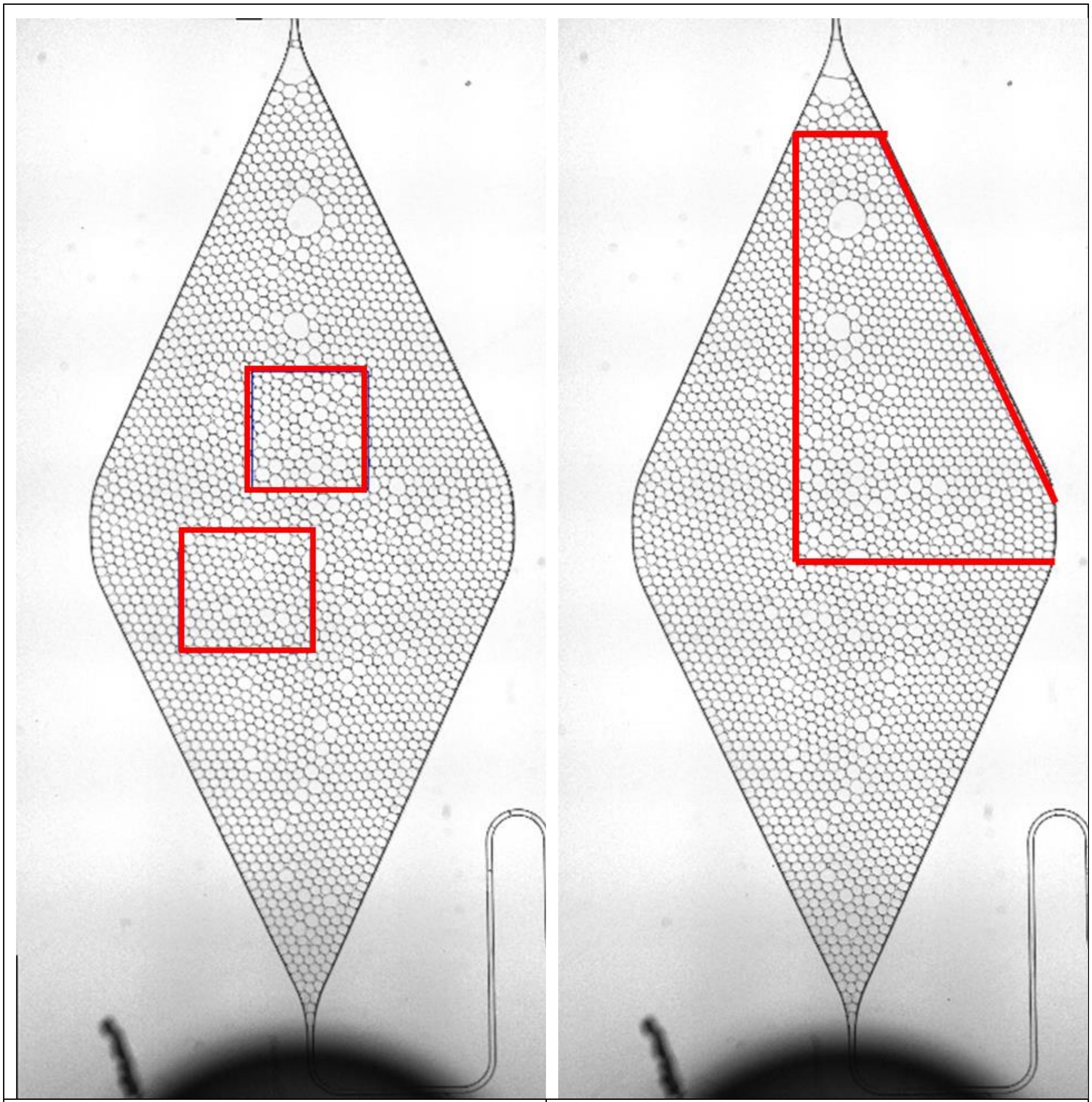

a. Two cropped regions covering 212 droplets.

b. Cropped region covering 550 droplets.

Figure S6 Cropped regions marked in the first image of the first dataset at t = 30 minutes of experiment B.

Image analysis

The first step after preprocessing the images was to identify the droplets, which was done by using the find Contours operation in the OpenCV-Python library[15]. Additional limitations were introduced to check if the detected contours were of circular shape. Based on the areas of the identified droplets, it was necessary to statistically identify and remove the droplets that were outliers. After testing the data using the q-q plot[20] and the Shapiro–Wilk test[21] to check its normality, it was determined that the data was not normally distributed, the 15th and the 95th percentile were determined to be the limits of valid data in our assessment. Afterwards, the remaining droplets were masked on the binarized image to remove the background to allow for a better analysis of the formed crystals. Finally, the find Contours operation was used again in the final image to determine the number of formed crystals. The final analysis for the cropped region # 1 at t = 30 minutes can be seen in **Figure S5**, where the formed crystals are identified and numbered.

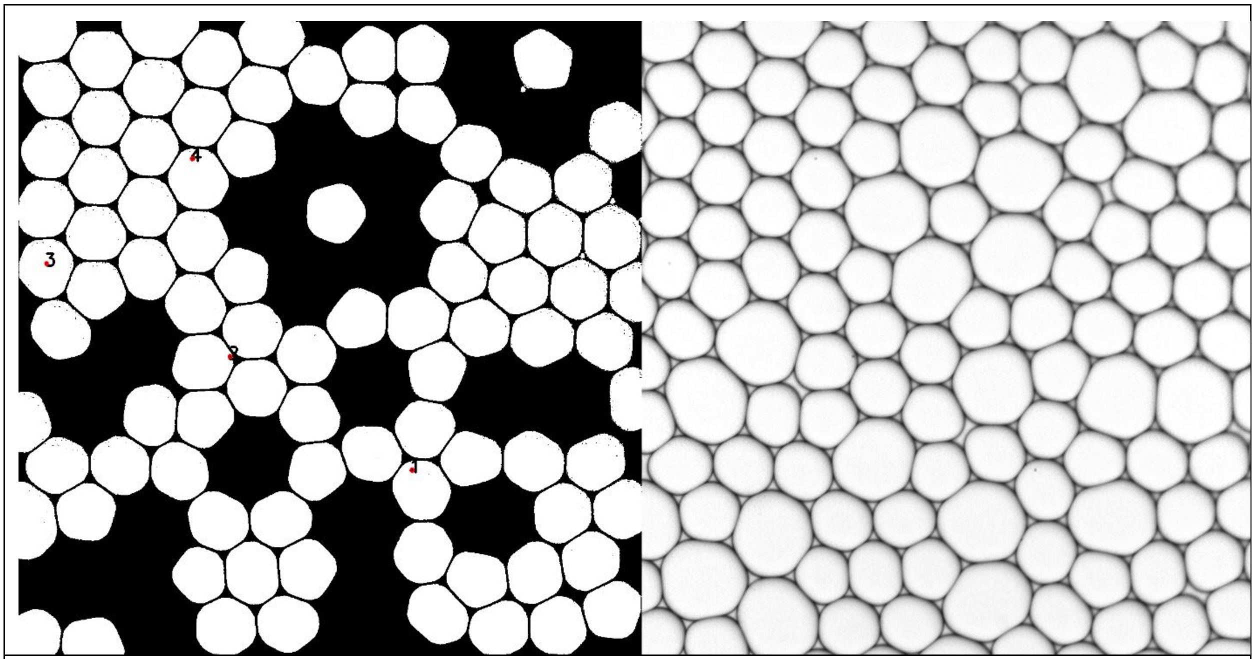

Figure S7 Final analysis of cropped region # 1 at t = 30 minutes.

Supplementary References

1 Scherer, G. W. Crystallization in pores. *Cement Concr. Res.* **242**, 1347-1358 doi: https://doi.org/10.1016/S0008-8846(99)00002-2 (1999).

2 Flatt, R. J. Salt damage in porous materials: how high supersaturations are generated. *J. Crys. Growth* **242**, 435-454 doi: https://doi.org/10.1016/S0022-0248(02)01429-X (2002).

3 Rijniers, L. A., Huinink, H. P., Pel, L. & Kopinga, K. Experimental evidence of crystallization pressure inside porous media. *Phys. Rev. Letters* **94**, 75503 doi: https://doi.org/10.1103/PhysRevLett.94.075503 (2005).

4 Emmanuel, S. & Berkowitz, B. Effects of pore-size controlled solubility on reactive transport in heterogeneous rock. *Geophys. Res. Letters* **34**, L06404, doi: https://doi.org/10.1029/2006GL028962 (2007).

5 Nielsen, A. E. Nucleation in aqueous solution. *J. Phys. Chem. Solids* **1**, 419-426 doi: https://doi.org/10.1145/1465611.1465667 (1967).

6 Schindler, P. W. Heterogeneous equilibria involving oxides, hydroxides, carbonates, and hydroxides carbonates. Equilibrium concepts in natural water systems. *Adv. Chem. Ser.* **67**, 197-221 doi: https://pubs.acs.org/doi/10.1021/ba-1967-0067.ch009 (1967).

7 Prieto, M. Nucleation and supersaturation in porous media (revisited). *Mineral. Mag.* **78**, 1437-1447 doi: https://doi.org/10.1180/minmag.2014.078.6.11 (2014).

8 Poonoosamy, J. *et al.* A microfluidic experiment and pore scale modelling diagnostics for assessing mineral precipitation and dissolution in confined spaces. *Chem. Geol.* **528**, 119264 doi: https://doi.org/10.1016/j.chemgeo.2019.07.039 (2019).

9 Dirany, M. *et al.* Chemical modification of PDMS surface without impacting the viscoelasticity: Model systems for a better understanding of elastomer/elastomer adhesion and friction. *Colloids Surf. A* **468**, 174-183 doi: https://doi.org/10.1016/j.colsurfa.2014.12.036 (2015).

10 Kulik, D. A. *et al.* GEM-Selektor geochemical modeling package: Revised algorithm and GEMS3K numerical kernel for coupled simulation codes. *Comput. Geosci.* **17**, 1-24, doi: https://link.springer.com/article/10.1007/s10596-012-9310-6 (2013).

11 Poonoosamy, J. *et al.* Barite precipitation following celestite dissolution in a porous medium: a SEM/BSE and micro XRF/XRD study. *Geochim. Cosmochim. Acta.* **182**, 131-144 doi: https://doi.org/10.1016/j.gca.2016.03.011 (2016).

12 Poonoosamy, J. *et al.* A lab-on-a-chip approach integrating in-situ characterization and reactive transport modelling diagnostics to unravel (Ba,Sr)SO_4 oscillatory zoning. *Sci. Rep.* **11**, 23678 doi: https://www.nature.com/articles/s41598-021-02840-9 (2021).

13 Dos Santos, E. C., Ladosz, A., Maggioni, G. M., von Rohr, P. R. & Mazzotti, M. Characterization of shapes and volumes of droplets generated in PDMS T-junctions to study nucleation. *Chem. Eng. Res. Des.* **138**, 444-457 doi: https://doi.org/10.1016/j.cherd.2018.09.001 (2018).

14 Van Rossum, Drake Jr, F. L. Python tutorial (Vol. 620). Amsterdam, The Netherlands: Centrum voor Wiskunde en Informatica (1995).

15 Bradski, G. The OpenCV Library. Dr. Dobb's Journal of Software Tools for the Professional Programmer 25, no. 11 (2000).

16 Harris, C. R. *et al.* Array programming with NumPy. *Nature* **585**, 357-362 doi:https://www.nature.com/articles/s41586-020-2649-2 (2020).
17 McKinney, W. Data structures for statistical computing in python. *Proc. of the 9th Python in Science Conf.*, 51-56 (2010).
18 Buades, A., Coll, B. & Morel, J. M. Non-local means denoising. *Image Processing On Line* **1**, 208-212 doi; https://www.ipol.im/pub/art/2011/bcm_nlm/?utm_source=doi (2011).
19 Gonzalez, R. C. Digital image processing. Pearson education India (2009).
20 Kaltenbach, H.-M. A concise guide to statistics. Springer, Berlin, Heidelberg (2012).
21 Shapiro, S. S. & Wilk, M. B. An analysis of variance test for normality (complete samples). *Biometrika* **52**, 591-611 doi:https://doi.org/10.2307/2333709 (1965).
